# Obstetrics

**Published:** 1876-06

**Authors:** 


### Summary of Progress in the Medical Sciences.

### I. Obstetrics.

1. *Prophylactic Measures against Puerperal Fever.* Bischoff. (Correspond. *Bl. f. Schweizer Aerzte ; Allg. Med. Central Ztg.*, 13, 1876.)

Prof. Bischoff, of Basil, considers puerperal fever as a traumatic fever which is very changeable in its symptoms. No puerperal fever without lesion and infection. And the usual locality of infection, he thinks, is n t the placental site but the cervical canal, and especially the external os uteri ; the frequent lacerations at these places, as also the lesions at the site of the urethral orifice, during the act of parturition, must be looked at as the usual starting points for the puerperal fever. The infection sometimes is occasioned by foul amniotic fluid or by the lochia rendered septic by decomposed coagula, shreds of the amnion or pieces of the placenta. But more frequently the septic virus is introduced from without by the unclean instruments or hands, or even by the entrance of foul air. Besides, the professor recognizes in some persons a greater predisposition to a local infection of wounds.

B.'s safeguard against puerperal fever is carbolic acid, which after a faithful trial of more than seven years he has good reasons to recommend most heartily. He employs it in a regular antiseptic treatment just as urgeons do in operative cases. His aim being to remove all noxious. matter before the genitals are wounded, and to prevent any infectious matter from entering these parts after they have received some lesion. Prof. B. enforced the following plan of antiseptic treatment in his lying-in hospital at Basil: At the commencement of labor pains the woman receives a bath and a vaginal injection of a lukewarm aqueous solution of carbolic acid (strength two per cent.) And these injections are repeated every two hours in all cases of protracted labor, of artificial premature labor, and of dead fœtus. Before making an exploration or operation the hands and instruments must be washed in a three per cent. solution of carbolic acid and lubricated with a carbolized glycerine ointment, or a carbolized oil containing 10 per cent. of carbolic acid. Immediately after the delivery the external parts are examined and all wounded places are touched with the carbolized oil. Lacerated wounds of the mucous membrane of the vagina, and those frequent, though superficial, rents near the orifice of the urethra, are then united at once by means of fine catgut sutures ; larger lacerations of the perineum are closed by silver wire, and these sutures are left undisturbed until the woman can get up, *i. e.*, for two weeks. And it is a very rare thing, then, not to find these lesions healed by first intention. After all the wounds are closed, a pledget of charpie,well saturated with carbolized oil, is put in the introitus vaginæ, to be renewed as often as it drops out, *i. e.*, after urinating and vaginal injections. Besides this, a second and larger pledget of carbolized charpie is laid over the external fissures or cuts. And in all cases, from six to twelve hours after delivery a vaginal injection with a two per cent. solution of carbolic acid is made, and afterward repeated two or three times daily; and if any coagula or pieces of the placenta are retained, the uterine cavity is washed out also by injections of carbolized water. These injections have never occasioned any untoward symptoms or had any poisonous effect.

As to the effectiveness of his prophylactic treatment B. gives the following statistics, concerning which he states, that he could not get the nurses to strictly comply with his directions, until two years ago, when the sisters of charity were employed as nurses.

| Time. | No. of confinements. | No. of deaths. | Per cent. | No. of puerp. fever. | Per cent. | Remarks. |
|---|---|---|---|---|---|---|
| 1862 to 1867 | 514 | 33 | 6.4 | 116 | 22 | Previous to antiseptic treatment. |
| 1868 | 83 | .. | 4.0 | 37 | 44.5 | Beginning of antiseptic treatment. |
| 1869 | 109 | .. | 6.4 | 41 | 37.6 | |
| 1870 | 80 | 0 | 0.0 | 13 | 22.3 | Better antiseptic treatment. |
| 1871 | 139 | 2 | 1.4 | 31 | 22.3 | |
| 1872 | 171 | 4 | 2.9 | 42 | 24.5 | Perfect antisepsis under the sisters of charity. |
| 1873 | 202 | 2 | 1.0 | 34 | 16.8 | |
| 1874 | 233 | 4 | 1.7 | 25 | 10.7 | |
| 1875 | 224 | 0 | 0.0 | A few | .... | |

2. *Subacute Cystitis following Parturition.* Dr. W. L. RICHARDSON, of the Boston Lying-in Hospital. (*The Boston Med. and Surg. Jour.*, Feb. 3, 1876.)

This condition is not mentioned in the works on Obstetrics of to-day. The reporter details four cases observed during the past three years. He suggests the possibility of cases of subacute cystitis being mistaken for metritis or circumscribed peritonitis. In all four of the cases reported, more or less protracted pressure by the fœtal head upon the bladder was exerted during the labor. In the severer cases—two out of the four—the invasion of the disease was announced by a chill; in one of these two cases, a relapse was initiated by a chill. Great dysuria in all cases was a marked feature and the symptom most complained of. Tenderness over the inferior abdomen was elicited upon pressure. Pain, nausea and vomiting were noticed in the severer cases. In all four of the cases there were mucus, pus, and, in only one case, blood, in the urine. The record of the pulse, temperature and breathing was singularly high in the evening, as compared with that in the morning. The temperature ran as high as 103° F., and the pulse as high as 124 beats per minute—these being the extremes noticed in either case. The case treated longest was under observation twenty-six days.

The only treatment adopted was poultices to relieve pain and tenderness; morphia, *per orem vel anum*, to quiet pain or dysuria; after subsidence of acute symptoms, the bladder was washed out with warm water or a weak solution of carbolic acid. In all cases the washing out was followed by a sudden subsidence of all the symptoms, that was very striking.

